# Determinants of diphtheria vaccination uptake during a mass immunization campaign in Mogadishu, Somalia: a cross-sectional study

**DOI:** 10.3389/fpubh.2026.1901409

**Published:** 2026-08-11

**Authors:** Mohamed Sharif Abdi, Yusuf Hared Abdi, Yakub Burhan Abdullahi, Nur Ahmed Dahir, Dahir Hussein sheikh Hassan, Laylo Abshir Ali, Ruqiya Abdullahi Nur, A/Asis Hassan Husein, Sharmake Gaiye Bashir, Naima Ibrahim Ahmed, Nuradin Abdullahi Sheikh Rashid, Ahmed Abdinasir Abdulle, Gallad Dahir Hassan

**Affiliations:** 1 Center for Health Research and Innovation, Somali National University, Mogadishu, Somalia; 2 Faculty of Health Science, Salaam University, Mogadishu, Somalia; 3 Faculty of Health Sciences and Tropical Medicine, Somali National University, Mogadishu, Somalia; 4 Faculty of Graduate Studies and Research, Somali National University, Mogadishu, Somalia

**Keywords:** diphtheria, internally displaced persons, outbreak-response immunization, Somalia, vaccination uptake, vaccine safety concerns

## Abstract

**Background:**

Diphtheria has re-emerged as a major public health threat in fragile and conflict-affected settings, where routine immunization systems are disrupted. Outbreak response vaccination campaigns are critical for rapid disease control; however, evidence of post-campaign vaccine uptake and its determinants among internally displaced populations remains limited. This study assessed diphtheria vaccination uptake and associated socio-demographic, behavioral, and communication-related factors following a mass immunization campaign in an internally displaced person (IDP) camp in Mogadishu, Somalia.

**Methods:**

A community-based cross-sectional post-campaign survey was conducted with 384 caregivers residing in an IDP camp in Mogadishu. Data were collected using a structured interviewer-administered questionnaire implemented in KoboToolbox. Variables included caregiver characteristics, household income, diphtheria awareness, exposure to campaign phone messaging, and behavioral and social drivers of vaccinations. Descriptive statistics and multivariable logistic regression analyses were performed to identify the factors independently associated with campaign vaccination uptake.

**Results:**

Overall, 57.6% of children reported receiving diphtheria vaccination during the campaign. After adjustment, higher vaccine safety concern scores were independently associated with lower odds of vaccination (adjusted odds ratio [AOR], 0.72; 95% CI: 0.57–0.90). Caregivers with primary education had lower odds of vaccinating their children than those with no formal education (AOR = 0.36; 95% CI: 0.14–0.93). Inverse associations were observed for prior diphtheria awareness and the receipt of phone-based messages, likely reflecting targeted outreach to households with unvaccinated children rather than deterrent effects. Trust in health workers, perceived access, and social norms were not independently associated with uptake.

**Conclusion:**

Diphtheria vaccination coverage following an outbreak response campaign in an IDP setting was suboptimal. Vaccine safety concerns have emerged as the primary modifiable barrier to uptake, underscoring the need for safety-focused risk communication and strengthened interpersonal counseling during emergency immunization efforts in fragile and displacement-affected contexts.

## Introduction

Diphtheria, a vaccine-preventable disease caused by toxigenic strains of *Corynebacterium diphtheriae*, remains a persistent threat in settings in which routine immunization systems have been disrupted by conflict, political instability, or natural disasters (1, 2). Despite the availability of highly effective diphtheria-tetanus-pertussis (DTP)-containing vaccines, the resurgence of diphtheria has been documented extensively in fragile and conflict-affected states, particularly where routine vaccination coverage intersects with large-scale population displacement, overcrowding, and weakened disease surveillance infrastructure (1, 3–5). Recent outbreaks in Yemen, Nigeria, Bangladesh, and multiple African regions have underscored that these epidemics disproportionately affect vaccinated children and occur predominantly in low-income urban areas and displacement camps (3, 5–8). The case-fatality ratio remains alarmingly high, ranging from 5 to 13% across recent outbreaks; however, the majority of deaths occur among unvaccinated or incompletely vaccinated children, with studies from Yemen and Nigeria demonstrating that 46–69% of fatal cases involve children with no prior DTP immunization (5, 7, 9). While outbreak-response vaccination campaigns represent a critical emergency control strategy to rapidly interrupt transmission and protect vulnerable populations, empirical data on real-world vaccine uptake following such campaigns remains sparse, particularly in the most vulnerable settings where multiple barriers to vaccination converge (3, 10).

Internally displaced persons (IDPs) are among the populations most vulnerable to vaccine-preventable disease outbreaks because of overcrowding, population mobility, limited healthcare access, and disruptions to routine immunization services (11–14). In Somalia, approximately 2.9 million people reside in IDP settlements, where vaccination coverage remains substantially lower than that in the general population (11, 13). These conditions increase susceptibility to diphtheria outbreaks and highlight the importance of evaluating the effectiveness of outbreak-response vaccination campaigns among displaced populations (11–14).

Somalia continues to experience major challenges in routine immunization because of prolonged conflict, recurrent humanitarian crises, and limited health-system capacity (3, 15, 16). These factors have may be associated with persistently low vaccination coverage and recurrent outbreaks of vaccine-preventable diseases, including diphtheria (3, 15, 16). Between 2024 and 2025, Mogadishu experienced a substantial increase in reported diphtheria cases, prompting outbreak-response vaccination activities targeting high-risk populations, particularly those residing in IDP settlements (3, 10, 17). Despite these efforts, evidence regarding vaccine uptake following emergency immunization campaigns remains limited.

Vaccination uptake is influenced by multiple behavioral and social factors beyond vaccine availability alone. The World Health Organization Behavioral and Social Drivers (BeSD) framework identifies key domains affecting vaccination behavior, including perceptions of disease and vaccine safety, social influences, motivation, and practical access considerations (18). Previous studies have highlighted the importance of caregiver awareness, confidence in vaccine safety, trust in health workers, and effective communication strategies in shaping vaccine uptake, particularly in fragile and humanitarian settings (13, 18, 19). Existing evidence from humanitarian settings has largely focused on routine childhood immunization, whereas determinants of vaccine uptake during emergency outbreak-response campaigns remain poorly understood, particularly among displaced populations (13, 14, 20).

Despite the increasing burden of diphtheria in Somalia and the implementation of outbreak-response vaccination campaigns, evidence regarding post-campaign vaccine uptake and its determinants among internally displaced populations remains scarce. Therefore, this study aimed to assess diphtheria vaccination uptake and identify socio-demographic, behavioral, and communication-related factors associated with child non-vaccination during the campaign among caregivers residing in an IDP camp in Mogadishu, Somalia.

## Methods

### Study design and setting

A community-based cross-sectional post-campaign assessment was conducted in Mogadishu, Somalia, following the most recent outbreak response diphtheria vaccination campaign. The study was implemented in an internally displaced persons (IDP) camp located in Gubadley district, which was purposively selected because it was among the districts reporting a high diphtheria outbreak burden and hosting large, densely populated IDP settlements. These conditions increased vulnerability to vaccine-preventable disease transmission and posed operational challenges for immunization delivery. The survey targeted caregivers residing in selected camps during the campaign period.

### Study population and eligibility criteria

The study population comprised adult caregivers (e.g., mothers, fathers, or other guardians) living in the selected IDP camp who were responsible for a child eligible for the diphtheria campaign vaccination. Caregivers were eligible if they resided in the camp during the campaign period and provided informed consent. Caregivers who were unable to communicate due to severe illness or who did not have a child in the eligible age group were excluded.

Only one caregiver–child pair was enrolled per household. Where a caregiver was responsible for more than one eligible child, a single index child was selected using a simple lottery method to avoid clustering of observations within households.

### Sampling strategy and sample size determination

A stratified systematic sampling strategy was used to select study participants. The internally displaced persons (IDP) camp was divided into enumeration areas (EAs), also referred to as administrative blocks/zones, which served as strata to ensure proportional representation across the study setting. Updated household lists obtained from camp administrators indicated a total number of households distributed across these strata.

For illustration of the sampling approach, each stratum (block/EA) was treated independently. In a typical allocation scenario, for example, five administrative blocks (Block A–E) may be used as strata. In such a case, the total sample is proportionally allocated to each stratum based on the number of households per block (e.g., Block A = 90, Block B = 80, Block C = 75, Block D = 70, Block E = 69 for a total of 384 households).

The sampling interval (*k*) was calculated separately for each stratum by dividing the total number of households in each block/EA by the allocated sample size. This produced a stratum-specific interval typically ranging between *k* = 5 and *k* = 7. A simple lottery method was used to select a random starting point between 1 and k, after which every kth household was selected systematically.

Within each selected household, one eligible caregiver was randomly selected using a lottery method when more than one eligible respondent was present. Each selected household was visited up to two times in case of absence. Households that remained unavailable or declined participation after two visits were classified as non-respondent and replaced by the next household in the sampling sequence.

The minimum sample size was determined using the single population proportion formula, assuming 50% vaccine uptake (due to lack of prior estimates in similar settings), a 95% confidence level (*Z* = 1.96), and a 5% margin of error (*d* = 0.05), yielding a final required sample of 384 households. Accordingly, one caregiver–child pair per household resulted in 384 caregiver respondents and 384 index children.

### Data collection tools and procedures

Data were collected electronically using KoboToolbox, allowing real-time data entry, skip logic, and automated quality control. A structured interviewer-administered questionnaire was used to assess diphtheria vaccination uptake and associated sociodemographic, behavioral, and communication-related factors.

The questionnaire captured caregiver demographics, household socioeconomic indicators, awareness of diphtheria, exposure to campaign communication (including recorded phone calls and messages), perceived credibility of messages, and behavioral determinants of vaccination such as trust, safety concerns, access, social norms, and preferred communication channels.

Data collectors received standardized training on study objectives, interviewing techniques, ethical considerations, and field procedures. The tool was pretested in a similar IDP setting outside the study area, and minor modifications were made for clarity and cultural appropriateness. Data were exported for cleaning and analysis. Statistical analysis was conducted using Stata software.

The instrument was developed based on the WHO Behavioral and Social Drivers (BeSD) of vaccination framework.

### Measurement of variables

The primary outcome was diphtheria vaccination uptake during the campaign (Yes/No). Vaccination status was verified using vaccination cards or visible fingermark ink for 221 of the 384 children (57.6%). For the remaining children, vaccination status was based on caregiver self-report. In cases of discordance between caregiver report and documented evidence (*n* = 12), card/fingermark verification was used as the final classification. A sensitivity analysis restricted to verified cases was conducted to assess potential misclassification bias.

Key explanatory variables included caregiver relationship to the child, education level, household income, awareness of diphtheria, exposure to phone-based messaging, and behavioral constructs (trust, vaccine safety concerns, access, social norms, and preferred information channels).

Each construct was measured using 3–5 Likert-scale items adapted from the WHO BeSD framework. Items were translated into Somali, back-translated into English, and refined during pretesting. Responses were scored on a 5-point Likert scale (1 = strongly disagree to 5 = strongly agree). Construct scores were computed as the mean of item responses.

Internal consistency was assessed using Cronbach’s alpha: Trust in health workers (*α* = 0.83), Vaccine safety concerns (*α* = 0.81), Access to vaccination services (*α* = 0.78), Perceived social norms (*α* = 0.76), and Preferred information channel (*α* = 0.74); all constructs demonstrated acceptable reliability (*α* ≥ 0.70).

### Data management and statistical analysis

Data were checked daily for completeness and consistency before being analyzed using Stata software. Descriptive statistics were used to summarize participant characteristics and study variables. Categorical variables were presented as frequencies and percentages, while Likert-scale variables were summarized using means and standard deviations.

Predictor variables with sparse categories (*n* < 10) were identified *a priori* and collapsed where necessary to ensure model stability. Bivariate logistic regression was conducted to assess crude associations between predictors and vaccination uptake. Variables with *p* < 0.20 in bivariate analysis, as well as conceptually relevant variables, were included in a multivariable logistic regression model.

Adjusted odds ratios (AORs) with 95% confidence intervals were reported. Statistical significance was set at *p* < 0.05. Model fit was assessed using likelihood ratio tests and pseudo-*R*^2^ statistics.

A complete-case analysis approach was used. Missing data accounted for 8 records (2.1%) and were excluded from regression analyses. This study followed the STROBE reporting guidelines for cross-sectional studies.

## Results

As shown in Table 1, the study population was predominantly composed of mothers (72.4%), followed by fathers (21.1%) and other caregivers (6.5%), indicating that child health decision-making within the IDP camp was mainly undertaken by women. Educational attainment was generally low, with 47.1% reporting no formal education and 37.2% having only Quranic education, while a small proportion had primary (13.0%) or secondary/higher education (2.6%). Socioeconomic conditions were highly constrained, as 83.3% of households reported a monthly income below USD 100.

**Table 1:** Socio-demographic characteristics and baseline awareness of respondents (*N* = 384).

Variable	*n* (%)
Respondent’s relationship to child	
Mother	278 (72.40)
Father	81 (21.09)
Grandmother/other relative	25 (6.51)
Highest level of education	
No formal education	181 (47.14)
Quranic school (Madrasa) only	143 (37.24)
Primary education	50 (13.02)
Secondary or higher education	10 (2.60)
Average monthly household income (USD)	
<100	320 (83.33)
100–200	56 (14.58)
201–400	8 (2.08)
Ever heard of diphtheria (Gawracat)	
Yes	288 (75.00)
No	96 (25.00)
Received recorded phone call/message in last 7 days	
Yes	269 (70.05)
No	115 (29.95)
Child received diphtheria vaccine during campaign	
Yes	221 (57.6)
No	163 (42.4)

Regarding health-related awareness, 75.0% of respondents reported having heard of diphtheria, while 70.1% reported exposure to phone-based campaign messages in the preceding week. These findings describe a population with high caregiving responsibility, low educational attainment, and substantial socioeconomic vulnerability, alongside moderate levels of disease awareness and communication exposure.

**Figure 1:**
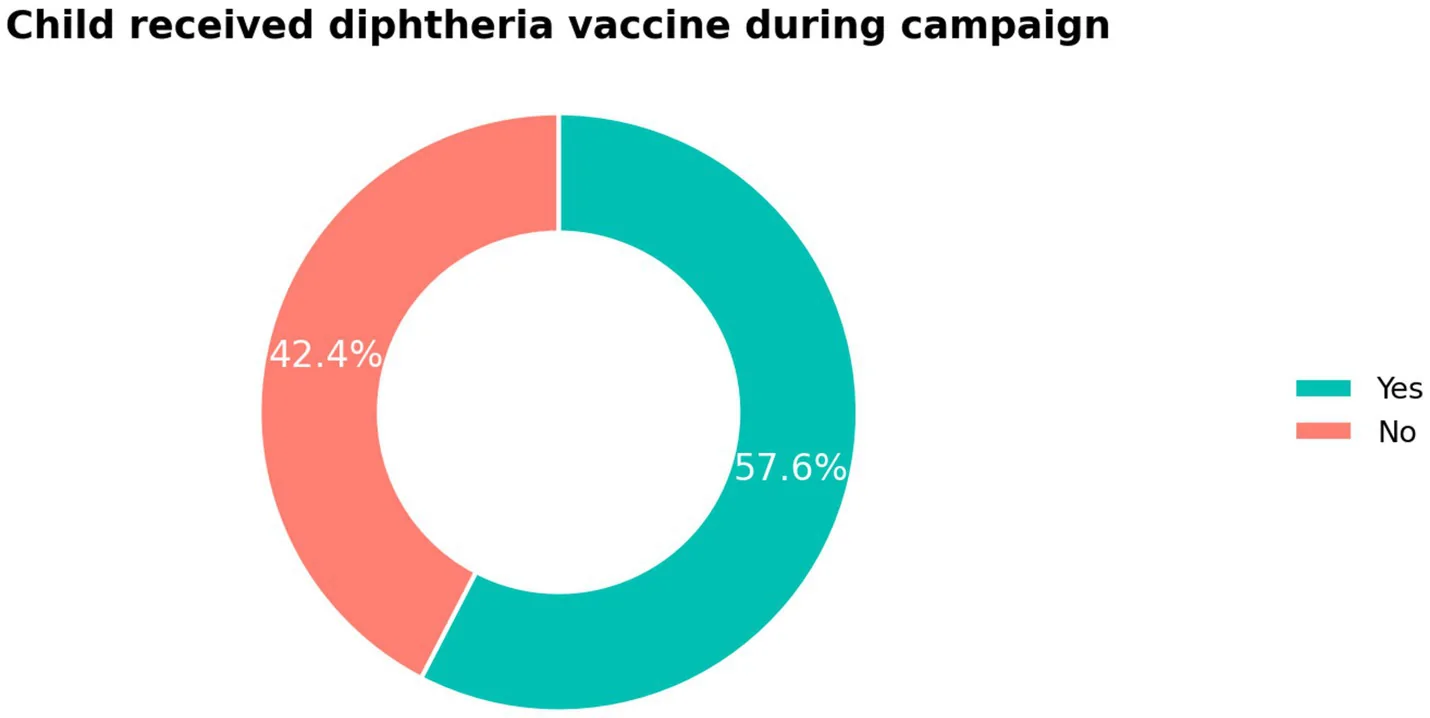
Proportion of children receiving diphtheria vaccination during a campaign.

Figure 1 shows that 57.6% of children received the diphtheria vaccine during the campaign, while 42.4% did not receive vaccination. This reflects the overall vaccination coverage achieved in the study population during the outbreak response campaign.

Among respondents who had heard of diphtheria (*n* = 288), perceived seriousness of the disease varied. Approximately 33.0% reported that diphtheria was not serious for a child, while 40.6% considered it somewhat serious. A further 26.4% perceived the disease as very serious or fatal.

Regarding perceived vaccine effectiveness, 54.2% of respondents believed that vaccines were very effective against diphtheria, 36.1% considered them somewhat effective, while 5.6% believed they were not effective and 4.2% reported not knowing their effectiveness (Table 2).

**Table 2:** Knowledge and perceptions regarding diphtheria and vaccination among respondents aware of the disease (*n* = 288).

Variable	*n* (%)
Perceived seriousness of diphtheria for a child	
Not serious	95 (32.99)
Somewhat serious	117 (40.63)
Very serious/fatal	76 (26.39)
Belief in effectiveness of vaccines against diphtheria	
Yes, very effective	156 (54.17)
Somewhat effective	104 (36.11)
No, not effective	16 (5.56)
I do not know	12 (4.17)

Among respondents who reported receiving a recorded phone message during the campaign (*n* = 269), 49.4% reported listening to the full message, while 46.8% listened only partially, and 3.7% reported hanging up immediately.

In terms of perceived source credibility, 66.9% identified the message voice as a doctor or health worker, 13.4% as a government official, 4.1% as a religious leader, and 15.6% as an unknown person. Regarding influence on vaccination decisions, 50.2% reported that the message had a little influence, 42.8% reported no influence, while 7.1% indicated that the message was a major reason for vaccinating their child (Table 3).

**Table 3:** Exposure to mobile phone messaging and perceived source credibility among respondents receiving recorded messages (*n* = 269).

Variable	*n* (%)
Listened to the full message	
Yes, fully	133 (49.44)
No, only part of it	126 (46.84)
No, hung up immediately	10 (3.72)
Perceived identity of the message voice (source credibility)	
Doctor/health worker	180 (66.91)
Government official	36 (13.38)
Religious leader	11 (4.09)
Unknown person	42 (15.61)
Influence of phone message on decision to vaccinate child	
A little	135 (50.19)
A lot (main reason for vaccination)	19 (7.06)
Not at all	115 (42.75)

As shown in Table 4, the mean scores for behavioral and social drivers (BeSD) of vaccination indicate generally neutral perceptions among respondents. Trust in health workers (mean = 2.98, SD = 1.63), perceived access to vaccination services (mean = 2.99, SD = 1.63), and perceived social norms (mean = 2.96, SD = 1.63) were all close to the midpoint of the Likert scale.

**Table 4:** Mean scores of trust, safety concerns, access, social norms, and preferred information channel (*N* = 384).

Construct	Mean	SD
Trust in health workers	2.98	1.63
Vaccine safety concern	3.04	1.54
Access to vaccination services	2.99	1.63
Perceived social norms	2.96	1.63
Preference for mobile phone calls	2.74	1.33

Likert scale: 1 = strongly disagree, 2 = disagree, 3 = neutral, 4 = agree, and 5 = strongly agree.

Vaccine safety concern recorded a slightly higher mean score (mean = 3.04, SD = 1.54), suggesting relatively greater concern compared to other constructs, while preference for receiving health information via mobile phone calls had the lowest mean score (mean = 2.74, SD = 1.33). The relatively large standard deviations across all constructs indicate considerable variability in responses among caregivers.

**Figure 2:**
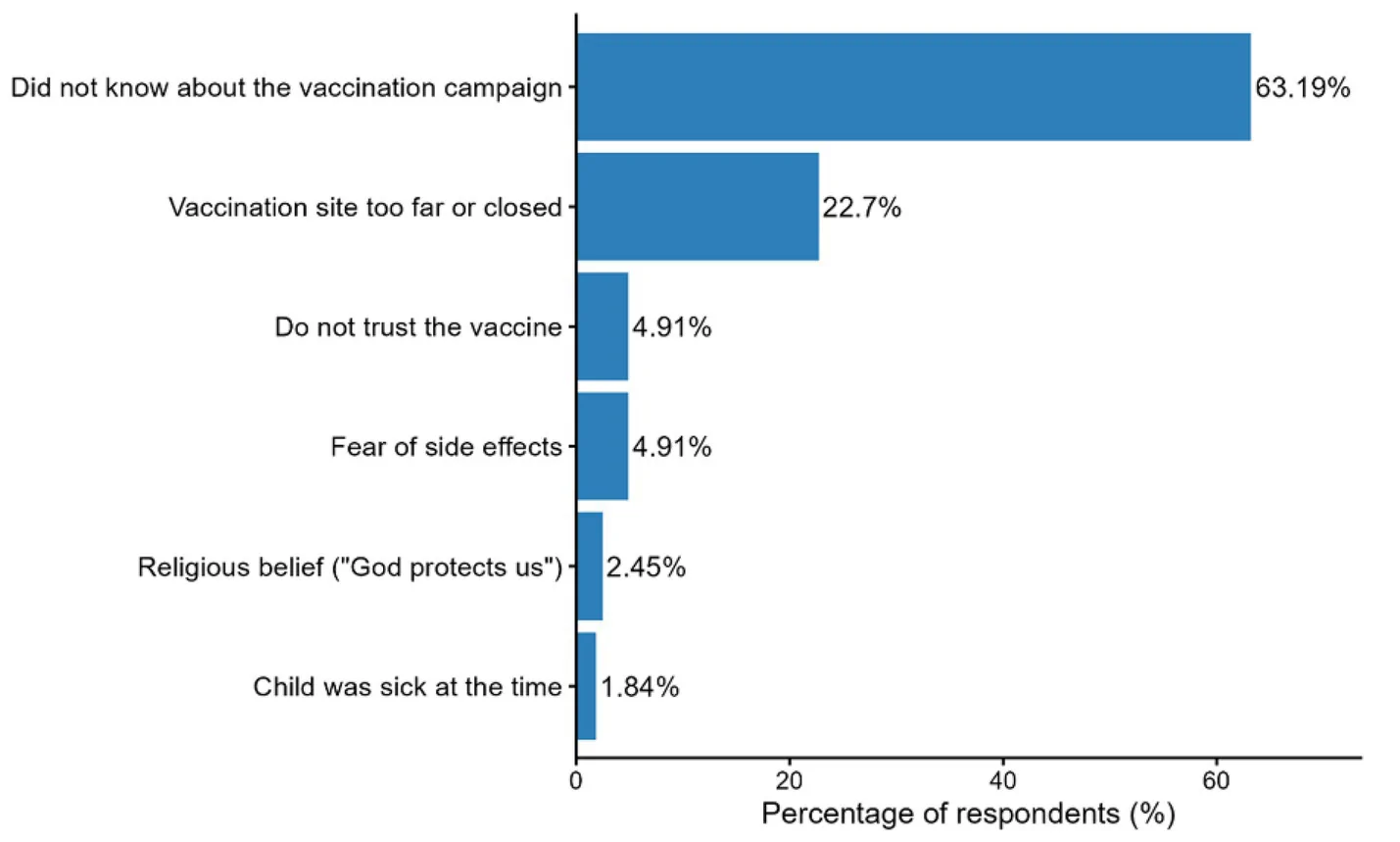
Reported barriers to diphtheria vaccination among caregivers of unvaccinated children (*n* = 163).

As shown in Figure 2, non-vaccination was driven primarily by structural and informational barriers rather than deliberate refusal. Lack of campaign awareness (63.19%) and poor site accessibility (22.70%) together accounted for roughly 86% of reported reasons (*n* = 163), whereas vaccine distrust (4.91%), fear of side effects (4.91%), religious beliefs (2.45%), and child illness (1.84%) were minor contributors. This indicates that improving coverage depends more on stronger community sensitization and service accessibility than on addressing vaccine hesitancy.

Bivariate and multivariable logistic regression analyses identified several factors associated with childhood non-vaccination during the diphtheria vaccination campaign (Table 5). In the adjusted model, caregivers with primary education had lower odds of having an unvaccinated child than those with no formal education (AOR = 0.36, 95% CI: 0.14–0.93; *p* = 0.036). Similarly, caregivers who had heard of diphtheria (AOR = 0.21, 95% CI: 0.10–0.41; *p* < 0.001) and those who received phone messages about the campaign (AOR = 0.18, 95% CI: 0.10–0.33; *p* < 0.001) had significantly lower odds of child non-vaccination. Among the psychosocial measures, each one-unit increase in the safety concern score was associated with a 28% reduction in the odds of child non-vaccination (AOR = 0.72, 95% CI: 0.57–0.90; *p* = 0.004). Caregiver relationship, household income, Quranic and secondary or higher education, trust, access, social norms, and information preference scores were not significantly associated with child non-vaccination after adjustment.

**Table 5:** Factors associated with childhood non-vaccination during the Diphtheria vaccination campaign (*N* = 384).

Predictor	COR (95% CI)	*p*-value	AOR (95% CI)	*p*-value
Respondent relationship to child				
Mother vs. father (ref)	1.21 (0.73–2.00)	0.467	0.97 (0.51–1.83)	0.923
Grandmother/other vs. father (ref)	1.75 (0.71–4.31)	0.226	0.76 (0.22–2.70)	0.676
Education level				
Primary vs. no formal education (ref)	0.30 (0.15–0.61)	0.001	0.36 (0.14–0.93)	0.036
Quranic vs. no formal education (ref)	0.61 (0.39–0.95)	0.029	0.62 (0.34–1.14)	0.127
Secondary+ vs. no formal education (ref)	0.24 (0.05–1.14)	0.073	0.23 (0.03–1.77)	0.157
Household income				
<100 USD vs. 100–200 USD (ref)	1.53 (0.85–2.78)	0.159	0.83 (0.38–1.82)	0.644
201–400 USD vs. 100–200 USD (ref)	1.17 (0.25–5.42)	0.842	1.60 (0.22–11.81)	0.645
Heard of diphtheria (yes vs. no)	0.10 (0.05–0.17)	<0.001	0.21 (0.10–0.41)	<0.001
Received phone message (yes vs. no)	0.10 (0.06–0.16)	<0.001	0.18 (0.10–0.33)	<0.001
Trust score (per unit increase)	1.08 (0.96–1.23)	0.209	0.95 (0.77–1.17)	0.612
Safety concern score (per unit increase)	0.94 (0.82–1.07)	0.356	0.72 (0.57–0.90)	0.004
Access score (per unit increase)	1.12 (0.99–1.27)	0.074	1.10 (0.88–1.37)	0.407
Social norm score (per unit increase)	1.07 (0.94–1.21)	0.317	0.99 (0.79–1.24)	0.943
Information preference score (per unit increase)	1.07 (0.92–1.25)	0.373	0.99 (0.79–1.24)	0.950

## Discussion

This post-campaign assessment conducted in an internally displaced persons camp in Mogadishu revealed that just over half of eligible children received diphtheria vaccination during an outbreak-response campaign, reflecting suboptimal coverage despite emergency mobilization efforts in a high-burden setting. Following multivariable adjustment, vaccine safety concerns emerged as the most consistent independent factor associated with child non-vaccination during the campaign, while sociodemographic factors, including caregiver relationships and household income, showed no significant associations. The finding that caregivers with primary education had lower odds of having an unvaccinated child than those with no formal education, coupled with inverse associations between child non-vaccination and both prior diphtheria awareness and phone-based message receipt, underscores the complexity of behavioral factor associated with in displacement-affected populations and challenges simplistic assumptions about knowledge, communication exposure, and vaccine acceptance during humanitarian emergencies.

The non-linear relationship observed between educational attainment and vaccination uptake warrants cautious interpretation, particularly the finding that caregivers with primary education had significantly lower odds of vaccinating their children than those with no formal schooling. However, this finding should be interpreted cautiously because only the primary education category reached statistical significance, the confidence interval was relatively wide, and no consistent dose–response relationship was observed across increasing educational levels.

Although this pattern differs from findings reported in some previous studies, it may represent a chance finding, residual confounding, or contextual characteristics unique to this IDP camp, additional studies are needed to determine whether this association is reproducible in other humanitarian settings (21). Studies conducted among internally displaced persons in Northeast Nigeria have similarly documented that educational level does not predict vaccine acceptance in straightforward ways, with complex interactions between formal education, traditional beliefs, access to health information, and perceptions of disease severity shaping immunization decisions (22). Research examining vaccination uptake among refugees and migrants across diverse humanitarian settings has found inconsistent associations between education and vaccine acceptance, with some contexts showing positive associations, others showing null effects, and still others consistent with the present findings showing inverse patterns among specific educational strata (23). Rather than reflecting a causal effect of education itself, these findings likely capture contextual factors that are systematically associated with educational attainment within this specific IDP setting, such as differential targeting of campaign messaging, heterogeneity in information sources accessed by educational groups, competing health priorities, or variation in risk perception. It is essential to avoid normative judgments and instead recognize that the relationship between education and vaccination behavior is context-dependent and shaped by the intersection of structural constraints, information environments, trust dynamics, and the unique vulnerabilities that characterize protracted displacement.

Vaccine safety concerns constituted the most robust independent associated with of reduced child non-vaccination in this study, with each unit increase in the safety concern score associated with lower odds of child non-vaccination. Even after controlling for awareness, communication exposure, trust, and access. This finding situates vaccine hesitancy within the broader literature on behavioral and social drivers of immunization in fragile and humanitarian settings, where fear of adverse events is amplified by the limited availability of follow-up care, weak pharmacovigilance systems, and past experiences of inadequate health service responsiveness (14, 24). Among displaced populations residing in camps with minimal healthcare infrastructure and uncertain access to emergency medical treatment, concerns about vaccine-related harm reflect rational risk aversion rather than ideological opposition to immunization. Studies conducted among internally displaced persons in conflict-affected settings have consistently identified vaccine safety perceptions as a primary factor associated with of uptake, with caregivers expressing fear of immediate adverse reactions, as well as the absence of reliable mechanisms to seek help if complications occur (21, 22). This dynamic is further exacerbated in outbreak-response campaigns, where rapid vaccine deployment, compressed timelines, limited opportunities for interpersonal counseling, and reliance on transient vaccination teams may heighten uncertainty and erode confidence in vaccine safety. The critical implication is that outbreak-response vaccination strategies in displacement settings must prioritize reassurance-focused risk communication that explicitly addresses safety concerns, transparently acknowledge the possibility of minor side effects while contextualizing their frequency and severity, provide clear guidance on how caregivers can access follow-up care if needed, and leverage trusted intermediaries such as community health workers and religious leaders to deliver credible, empathetic messaging that validates caregivers’ concerns while reinforcing the protective benefits of vaccination.

The inverse associations observed between child non-vaccination and both prior awareness of diphtheria and the receipt of phone-based messages require methodologically informed interpretation to avoid erroneous conclusions that information or communication discourages vaccination. Importantly, because exposure to awareness activities and phone-based communication was measured after the campaign in the same survey as vaccination status, the temporal sequence between communication exposure and vaccination could not be established. Consequently, these associations should not be interpreted as evidence that awareness or mobile messaging reduced vaccination uptake. Rather, they are more plausibly explained by reverse causation or targeted communication strategies in which campaign teams preferentially contacted households whose children had not yet been vaccinated. These findings almost certainly reflect the programmatic targeting and timing effects inherent in cross-sectional post-campaign assessments, whereby households with unvaccinated children were preferentially contacted during the later phases of the campaign, as health teams intensified outreach efforts to reach defaulters and close coverage gaps. Observational studies of vaccination campaigns are inherently susceptible to selection bias and reverse causation, particularly when communication exposure is measured retrospectively, as participants who did not vaccinate may have been disproportionately targeted for additional follow-up messages, phone calls, or home visits precisely because their initial nonparticipation was documented (25). Methodological literature on causal inference in vaccine safety and effectiveness research has consistently cautioned against interpreting temporally associated events, such as message receipt following non-vaccination, as indicative of causation without rigorous assessment of confounding, selection mechanisms, and temporal ordering (25, 26). The present findings do not imply that diphtheria awareness or mobile messaging discourages vaccine uptake; rather, they highlight the operational complexity of emergency immunization campaigns, where the real-time identification of non-responders triggers targeted communication strategies that create spurious inverse associations in post-campaign cross-sectional data. These results underscore the importance of integrating process evaluation and programmatic monitoring data to disentangle the effects of communication interventions from the selection processes that determine who receives intensified outreach and reinforce the need for prospective or quasi-experimental designs when evaluating the causal impact of risk communication strategies during outbreak-response vaccination campaigns.

The absence of independent associations with child non-vaccination and trust in health workers, perceived access to services, and social norms following multivariable adjustment suggests that, while these factors are necessary preconditions for campaign implementation, they may be insufficient on their own to overcome salient behavioral potential barrier such as vaccine safety concerns. Trust in healthcare providers and perceived accessibility represent foundational elements of functional immunization systems; however, research examining pathways linking trust to vaccine uptake has demonstrated that trust often operates indirectly through its was associated with on perceptions of vaccine safety, confidence in vaccine effectiveness, and willingness to accept provider recommendations (27, 28). In settings where vaccine safety concerns are pronounced, high levels of trust and adequate physical access may not fully compensate for caregivers’ apprehension about potential harm, particularly when opportunities for personalized counseling and risk communication are limited during rapid campaign delivery. Studies conducted in humanitarian and conflict-affected contexts have similarly found that trust and access, although critical, are mediated by confidence in vaccine safety and perceived disease severity, with the strongest factor associated with of uptake being behavioral and perceptual factors rather than structural or relational factor associated with alone (22, 24). The wide variability observed in Likert scale scores for trust, access, and social norms further indicates substantial heterogeneity in caregivers’ attitudes within the camp, suggesting that population-level interventions must be complemented by tailored approaches that address the specific concerns of subgroups exhibiting lower confidence or heightened safety apprehension. Collectively, these findings emphasize that outbreak-response vaccination campaigns in IDP settings require a multi-level intervention strategy that strengthens both the structural foundations reliable vaccine supply, accessible service delivery points, and competent health workforce and the behavioral dimensions, including targeted risk communication, interpersonal counseling by trusted providers, and community engagement that proactively address safety concerns and build vaccine confidence.

Although these findings are derived from a single IDP camp in Mogadishu, they provide useful insights that may inform planning of outbreak-response vaccination campaigns in similar humanitarian settings. Their applicability to other IDP camps or regions should be interpreted cautiously because campaign implementation, population characteristics, and health-system contexts may differ. First, safety-focused messaging must be elevated as a central pillar of campaign communication, with materials and interpersonal counseling explicitly addressing common fears about adverse events, providing transparent information on vaccine safety profiles, and delineating pathways for caregivers to access medical support if side effects occur. Second, interpersonal communication by trained health workers and community health volunteers should be strengthened, as trusted intermediaries are uniquely positioned to deliver nuanced, empathetic risk communication that acknowledges caregivers’ concerns, while reinforcing the life-saving benefits of vaccination during outbreaks. Third, the timing, clarity, and targeting of mobile phone-based messaging should be optimized, with early dissemination of educational content prior to campaign initiation, to build awareness and confidence, rather than relying primarily on reactive outreach to households identified as non-responders. Fourth, outbreak-response campaigns must integrate rapid behavioral diagnostics, such as pre-campaign formative assessments, to identify prevalent concerns, trusted information sources, and potential barrier to uptake, to enable real-time adaptation of messaging and service delivery strategies. Importantly, these findings underscore that rapid vaccine delivery alone is insufficient; effective outbreak control in displacement settings require concurrent investment in demand generation, trust building, and safety reassurance. These observations may also be relevant to other outbreak-response vaccination programs implemented under comparable humanitarian conditions, although confirmation in additional settings is required.

### Strengths and limitations

This study has several strengths that enhance the validity and utility of its findings. First, it focuses on an under-studied population internally displaced persons residing in a camp setting who face compounded vulnerabilities, yet are rarely the subject of rigorous post-campaign vaccination assessments, thereby addressing a critical evidence gap in humanitarian immunization research. Second, the assessment was conducted immediately following a real outbreak-response campaign, capturing vaccination behavior under authentic emergency conditions rather than hypothetical scenarios or routine service delivery contexts. Third, the integration of behavioral constructs derived from the World Health Organization’s Behavioral and Social Drivers framework, including trust, safety concerns, social norms, and communication exposure, alongside sociodemographic variables, enables a comprehensive, theoretically grounded analysis of the factor associated with child non-vaccination during the campaign. However, several limitations must be acknowledged in order to guide the appropriate interpretation and application of the findings. The cross-sectional design precludes causal inference, as temporal associations between exposures and vaccination status cannot definitively establish directionality, and unmeasured confounding may bias the observed associations. This limitation is particularly relevant for the inverse associations observed between prior diphtheria awareness, phone-message exposure, and child non-vaccination. Because communication activities may have occurred after households were identified as unvaccinated during campaign implementation. Reliance on caregiver self-reports for vaccination status, although supplemented by card verification where available, introduces potential recall bias and social desirability bias, which may overestimate or underestimate true coverage. The single-camp setting, while providing depth of assessment, may limit generalizability to other IDP camps in Mogadishu or to displaced populations in different regions of Somalia with distinct demographic profiles, outbreak histories, and health system capacities. These limitations do not undermine the internal validity of the associations identified within this study population, but require cautious extrapolation of findings to other contexts and reinforce the need for multi-site, longitudinal assessments to validate and extend these results. Consequently, the findings should be interpreted as associations observed within this specific IDP camp rather than estimates that are directly generalizable to all internally displaced populations in Somalia or other humanitarian settings.

In this IDP camp, child non-vaccination during the diphtheria vaccination campaign was more strongly associated with caregivers’ perceptions of vaccine safety than with measured sociodemographic characteristics or perceived access. The inverse associations between child non-vaccination and both prior awareness of diphtheria and communication exposure most likely reflect the operational realities of targeted campaign outreach rather than deterrent effects of information, while the absence of independent associations for trust, access, and social norms suggests that these factors, although important, are insufficient to overcome safety-related concerns. This study provides evidence from one IDP camp that may help inform future outbreak-response vaccination campaigns in comparable humanitarian settings. These findings are relevant to Somalia’s ongoing efforts to strengthen outbreak preparedness and response, as well as broader humanitarian immunization programs in settings where conflict, displacement, and fragile health systems create persistent challenges to achieving high vaccination coverage.

## Data Availability

The raw data supporting the conclusions of this article will be made available by the authors, without undue reservation.
